# Exploring the Association Between Attention-Deficit/Hyperactivity Disorder and Essential Hypertension in a Pediatric Population

**DOI:** 10.3390/children13010107

**Published:** 2026-01-12

**Authors:** Eugene Merzon, May Poluksht, Shai Ashkenazi, Ehud Grossman, Eli Magen, Akim Geishin, Iris Manor, Abraham Weizman, Avivit Golan-Cohen, Shlomo Vinker, Ilan Green, Alexander Bershadsky, Ariel Israel

**Affiliations:** 1 Adelson School of Medicine, Ariel University, Ariel 4070000, Israel; may.poluksht@msmail.ariel.ac.il (M.P.); shaias@ariel.ac.il (S.A.); ehud.grossman@ariel.ac.il (E.G.); 2 Leumit Health Services, Tel Aviv-Yafo 6473817, Israelaisrael@leumit.co.il (A.I.); 3 Medicine Department A, Assuta Ashdod University Hospital, Ha-Refu’a St 7, Ashdod 7747629, Israel; 4 Faculty of Health Sciences, Ben-Gurion University, Beer-Sheba 8410501, Israel; 5 Geha Mental Health Center, Petah-Tikva 4910000, Israel; 6 Department of Psychiatry, Gray Faculty of Medical & Health Sciences, Tel Aviv University, Tel Aviv 6997801, Israel; 7 Laboratory of Molecular and Biological Psychiatry, Gray Faculty of Medical & Health Sciences, Tel Aviv University, Tel Aviv 6997801, Israel; 8 Department of Family Medicine, Gray Faculty of Medicine, Tel-Aviv University, Tel Aviv-Yafo 6997801, Israel; 9 Department of Epidemiology and Disease Prevention, Gray Faculty of Medical & Health Sciences, Tel Aviv University, Tel Aviv 6139001, Israel

**Keywords:** attention-deficit/hyperactivity disorder, neurodevelopmental disorders, hypertension, pediatric populations, children

## Abstract

**Highlights:**

**What are the main findings?**

- Children diagnosed with ADHD had a significantly higher long-term prevalence of essential hypertension compared to matched controls, with odds ratios ranging from 3.17 at 5 years to 1.92 at 20 years of follow-up.
- ADHD patients demonstrated greater use of antihypertensive medications, including calcium channel blockers, renin–angiotensin system blockers, and diuretics, indicating clinically meaningful hypertension requiring pharmacological management.

**What are the implications of the main findings?**

- These results underscore the need for regular cardiovascular monitoring in pediatric patients with ADHD to enable early detection and management of hypertension.
- The findings suggest potential shared biological and behavioral mechanisms between ADHD and hypertension, highlighting the importance of integrated care and further research to guide preventive strategies.

**Abstract:**

**Objective**: Current data on the association between attention-deficit/hyperactivity disorder (ADHD) and essential hypertension (EH) in pediatric populations are very limited, as most research has focused on adults. This study investigated the long-term prevalence of EH in Israeli youth aged 5–18 years with ADHD, examining also trends in antihypertensive medication use. **Methods**: A retrospective cohort study was conducted using data from Leumit Health Services. The ADHD cohort (N = 18,558) was compared in a 1:2 ratio to controls (N = 37,116), who were strictly matched for age, gender, birth year and quarter, socioeconomic status (SES), sectors, region, and cumulative years of LHS membership up to the index date. Diagnoses of ADHD and EH were identified using ICD-9/10 codes, depending on the year of diagnosis. Logistic regression analyses were used to assess the associations between ADHD, EH and the use of antihypertensive medications over a 20-year follow-up. **Results**: ADHD-diagnosed children had a higher prevalence of EH, with odds ratios (ORs) of 3.17 (95% CI: 1.46–7.16, *p* = 0.0017) at 5 years, 2.94 (95% CI: 1.45–6.09, *p* = 0.0013) at 10 years, and 1.92 (95% CI: 1.26–2.93, *p* = 0.0015) at 20 years. ADHD patients showed a greater use of antihypertensive medications, including calcium channel blockers (OR 1.85, 95% CI: 1.02–3.35, *p* = 0.035), renin angiotensin system blockers (OR 2.20, 95% CI: 1.15–4.25, *p* = 0.013), and diuretics (OR 1.77, 95% CI: 1.21–2.60, *p* = 0.0028). **Conclusions**: These findings highlight an association between ADHD diagnosis and EH, suggesting regular cardiovascular monitoring of children with ADHD. Further studies are needed to uncover the role of stimulant medications and shared biological and behavioral factors involved in the pathogenesis.

## 1. Introduction

Attention-Deficit/Hyperactivity Disorder (ADHD) is the most prevalent neurodevelopmental disorder in children, with an estimated global prevalence of 7.2% [1,2]. Characterized by persistent patterns of inattention, hyperactivity, and impulsiveness, ADHD can significantly impair social, academic, and behavioral functioning.

Mounting evidence has linked ADHD with increased risks of somatic comorbidities. A Swedish population study reported a threefold increase in obesity risk among individuals with ADHD, especially among genetically related individuals [3]. Additional research identified a higher ADHD prevalence among children with type 1 diabetes [4], and meta-analyses have shown associations with sleep-disordered breathing [5] and reduced life expectancy [6].

Essential Hypertension (EH), defined as persistently elevated blood pressure without an identified cause, affects approximately one-third of adults globally [7]. In children and adolescents under 19 years of age, the prevalence of EH is estimated at 4% [8]. The diagnosis of EH in children is challenging because blood pressure is sometimes not appropriately measured and the a need to rule out secondary hypertension. EH contributes to a wide range of cardiovascular morbidities, including coronary artery disease, heart failure, stroke, and chronic kidney disease [9,10].

Regarding ADHD and hypertension, previous studies examined the effects of stimulant treatment of adults with ADHD on heart rate and hypertension [11,12,13]. Stimulant medications (methylphenidate, amphetamines) are widely used for the treatment of ADHD, and as sympathomimetic agents, they increase heart rate and blood pressure [11]. However, ADHD may be associated with an increased rate of EH not only as an adverse effect of stimulant medications, but also via shared metabolic pathways or behavior, as it is associated with somatic comorbidities [3], systemic inflammation, oxidative stress and immune dysregulation [2]. Fuemmeler et al. documented associations between ADHD symptom severity, obesity and hypertension in young adults in an uncontrolled study [14].

These data highlight a significant knowledge gap regarding the association between ADHD and validated EH in children, as ADHD is usually diagnosed in school-aged children, and diagnosing EH in children and adolescents is of prime importance. Additionally, using a matched control group and conducting prolonged follow-up are crucial for properly exploring this topic. Therefore, considering the limited research investigating the specific association between ADHD and cardiovascular risk in pediatric populations, there is a clear need for further systematic study in this area. Our research question was whether associations exist between ADHD in children and EH and the use of antihypertensive medications. This study comprehensively investigated these topics, utilizing a population-based dataset with a prolonged follow-up period. By examining the prevalence of long-term hypertension and medication trends, the research aims to clarify the association of ADHD and hypertension in children.

## 2. Methods

**Study Design**: We conducted a population-based, retrospective cohort study within Leumit Health Services (LHS), a nationwide Health Maintenance Organization (HMO) in Israel serving 724,129 individuals throughout the study period. LHS maintains an extensive computerized database that is continuously updated with information on demographics, medical visits, laboratory tests, hospitalizations, and medication prescriptions. Moreover, prescription records dating back to 1998, including refills and purchases per patient, are accessible. All LHS members enjoy uniform general health insurance coverage and equitable access to healthcare services.

**Study Population and Definitions**: The study population consisted of all LHS members between 1 January 2002 and 30 November 2022, aged 5 to 18 years. The exposed group consisted of individuals with a confirmed diagnosis of ADHD based on ICD-9/10 codes (314.00–314.9), depending on the year of diagnosis. Controls consisted of LHS members during the same period without a diagnosis of ADHD, at a 1:2 ratio, individually matched for age, gender, birth year and quarter, socioeconomic status (SES), sectors, region, cumulative years of LHS membership up to the index date, and body mass index (BMI). Obesity was defined as a BMI ≥ the 95th percentile for age and sex, based on CDC international growth charts. In addition to demographic matching, we examined the prevalence of major chronic comorbidities in both groups, including bronchial asthma, diabetes mellitus, and epilepsy, to characterize the study population and assess potential confounding.

**The exposure**: The confirmed diagnosis of ADHD adhered to the criteria set forth by the Israeli Ministry of Health, in alignment with international evaluation standards [1]. Additionally, the diagnosing physician was required to be a senior specialist in the field of ADHD (child or adult psychiatrists, child or adult neurologists, or pediatricians and family physicians with certified ADHD training).

**The outcome**: All clinical diagnoses of essential hypertension were based on ICD-9 codes: 401—Hypertension, Essential. The diagnosis of EH in children was performed according to national guidelines, emphasizing the requirement for appropriate measurements of blood pressure in children and establishing the diagnosis by age- and sex-adjusted blood pressure percentiles. Because the timing of initiation of stimulant medications, which are often used to treat children with ADHD, and as sympathomimetic agents, may increase blood pressure, it is possible that the diagnosis of hypertension was made after the initiation of stimulant medications. Quantifying stimulant exposure in real-world settings is highly challenging. Many individuals use these medications intermittently, often on an as-needed basis—such as before exams or specific tasks—and may purchase a single prescription that lasts for months or even a year. This irregular usage pattern makes it difficult to accurately capture cumulative exposure or establish dose–response relationships using administrative data. As part of our quality assurance process, we conducted a manual review of medical records for a subset of cases to confirm the accuracy of ADHD and hypertension diagnoses.

For antihypertensive medications, ATC codes were recorded in the electronic medical records (EMR) of LHS. The diagnosis of essential hypertension was based on ICD-9 codes combined with prescription data for antihypertensive medications, ensuring identification of clinically managed cases. The dataset did not include detailed information on individual anti-hypertensive treatment regimens, such as dosage, titration, or whether the same medication was continued throughout the follow-up. Therefore, analyses reflect the presence of fulfilled prescriptions rather than longitudinal dose trajectories.

SES classification was based on the Israeli Central Bureau of Statistics system, featuring 20 sub-groups. Classifications one to three, four to six, seven to nine, and 10 to 20 signified very low, low, medium, and medium-high SES, respectively.

To explore potential lifestyle confounding, we extracted available data on physical activity from the electronic health records. Physical activity was categorized into five groups: none, occasional, 1–3 h weekly, >3 h weekly, and missing. This variable was included in descriptive analyses to assess distribution across the ADHD and control groups. Due to the high proportion of missing data, physical activity was not incorporated into the matching algorithm or regression models but was retained for transparency.

The meticulous matching of the control group with cases in terms of relevant variables (age, gender, ethnicity, region, SES and BMI) aimed to minimize potential confounding factors.

**Statistical Analysis**: We conducted all statistical analyses using R statistical software, rejecting the null hypothesis for two-sided values of *p* < 0.05. Socio-demographic characteristics were compared between the ADHD and non-ADHD control groups using the t-test for continuous variables and Fisher’s exact test for categorical variables. Logistic regression analysis was employed to assess the association between the outcome (the clinical diagnosis of essential hypertension) and the exposure (ADHD diagnosis), yielding odds ratios (ORs) and 95% confidence intervals (CIs). We used logistic regression models at predefined intervals (1, 5, 10, 15, and 20 years after ADHD diagnosis) rather than time-to-event methods because the dataset did not include precise dates of hypertension onset for all cases. While this method allowed us to estimate odds ratios at clinically meaningful horizons, it does not account for varying follow-up times and censoring, which may introduce bias. Only the year of diagnosis and prescription fulfillment were available, which precluded accurate modeling of event times and censoring. Our primary objective was to estimate the odds of having a documented diagnosis of essential hypertension at clinically relevant time points, rather than instantaneous risk. Logistic regression provided interpretable estimates for these fixed horizons. The intervals were selected to represent short-, medium-, and long-term clinical milestones: early childhood (1 year), mid-childhood/adolescence (5–10 years), late adolescence (15 years), and transition to young adulthood (20 years). These time frames align with pediatric follow-up practices and allow clinicians to contextualize risk across developmental stages. Individuals were included in each interval only if they maintained continuous LHS membership during that period, minimizing bias from differential follow-up. Participants who left the HMO before a given time point were excluded from that interval’s analysis. Missing BMI and socioeconomic status values were retained as separate “missing” categories in matching and regression models rather than imputed, to avoid introducing artificial variance. This approach ensured transparency and preserved the integrity of the matched design.

We used the Benjamini–Hochberg procedure to control the false discovery rate (FDR) for multiple tests. This method reduces the likelihood of type I errors (false positives) by adjusting *p*-values in a way that balances statistical rigor with the ability to detect true associations. Clinically, this approach ensures that reported differences in being diagnosed with EH are less likely to be due to chance, thereby improving the reliability of findings.

This study was based on anonymized data extracted from an electronic database; no direct interaction with human participants occurred, and therefore informed consent was not required. The study was conducted in accordance with ethical standards and approved by the Institutional Review Board (authorization number LEU-0005-22).

## 3. Results

### 3.1. Study Groups and Matching

A total of 18,558 children with ADHD diagnoses were identified and compared with a control group of 37,116 matched individuals, at a 1:2 ratio. Table 1 provides a detailed demographic and body mass index comparison between the two groups, demonstrating a very good match for age, gender, sector, SES, geographic region, and BMI. The prevalence of obesity at diagnosis was similar between groups: 301 children with ADHD (1.74%) and 554 controls (1.77%) (*p* = 0.829; OR = 1.09, 95% CI: 0.94–1.26), indicating no significant difference. Physical activity data were largely missing (≈88% in both groups), limiting their utility for adjustment. Among those with documented activity, distributions were similar between the ADHD and control groups, suggesting no major imbalance (Table 1).

In addition to demographic characteristics, we examined major chronic comorbidities in both groups. Bronchial asthma was more frequent among children with ADHD (4666; 25.1%) compared to controls (8393; 22.6%) (*p* = 0.001; OR = 1.15, 95% CI: 1.10–1.20). Diabetes mellitus showed similar prevalence in both groups (61 cases, 0.33% vs. 130 controls, 0.35%; *p* = 0.701; OR = 0.94, 95% CI: 0.68–1.28). Epilepsy was notably higher in the ADHD group (306 cases, 1.65%) compared to controls (294, 0.79%; *p* = 0.001; OR = 2.10, 95% CI: 1.78–2.48).

### 3.2. Essential Hypertension

**Figure 1:**
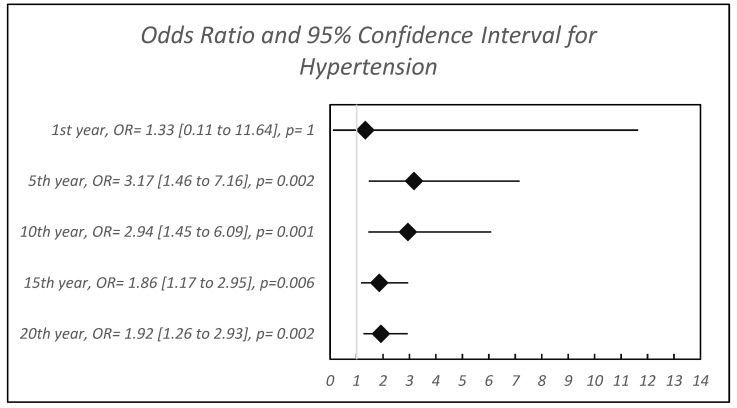
Odds Ratios and 95% Confidence Interval for being diagnosed with essential hypertension in children with ADHD.

Table 2 provides an overview of the prevalence of the diagnosis of essential hypertension in the study and control groups, followed by a forest plot that graphically displays the results (Figure 1). Although the overall prevalence of ES was very low, the ADHD group (N = 18,558) exhibited a significantly higher prevalence of hypertension compared to the control group (N = 37,116) over the various periods of follow-up.

Figure 1 illustrates the adjusted odds ratios for EH diagnosis among children with ADHD compared to matched controls across five follow-up intervals. While the relative risk is consistently elevated, the absolute prevalence remains very low (0.25% at 20 years), and the absolute difference was low, 0.13%. This means that, although the association is statistically significant based on robust epidemiologic data, the clinical impact in terms of absolute numbers is mild.

### 3.3. Antihypertensive Medication Use in ADHD and Control Groups over Time

Table 3, Table 4 and Table 5 summarize the use of three categories of antihypertensive medications—Calcium Channel Blockers (C08), Agents Acting on the Renin–Angiotensin System (C09), and Diuretics (C03), by the ADHD and control groups across various follow-up periods (1, 5, 10, 15, and 20 years). The doses of the medications were adjusted by age and weight. These data provide insight into treatment patterns in children with hypertension and controls, and suggest that cases of hypertension identified in the ADHD group were probably clinically meaningful, as they required pharmacological management. However, the overall prescription rates were extremely low (<0.4% even at 20 years) and the differences between the groups were also very low, reinforcing that these findings should be viewed in the context of rarity and interpreted as exploratory signals rather than definitive evidence of widespread clinical risk.

#### 3.3.1. Calcium Channel Blockers (C08)

Over the 20 years, the use of calcium channel blockers was consistently higher in the ADHD group compared to controls. However, statistical significance was only observed at the 15-year mark (*p* = 0.035, FDR-adjusted q = 0.071), with an OR of 1.85 [95% CI: 1.02–3.35]. By the 20th year, although the trend of increased use continued (OR 1.69), significance was not retained (*p* = 0.052, q = 0.101). Details are presented in Table 3.

**Table 3:** Calcium Channel Blockers Prescribed Following Initial Diagnosis of ADHD and Controls Over a 20-Year Follow-up Period (presented as number [%]).

C08 Calcium Channel Blockers	Case N = 18,558	Controls N = 37,116	OR (95%CI)	*p* Value	FDR BH
1st year after diagnosis	5 (0.027%)	4 (0.011%)	2.50 [0.54 to 12.60]	0.170	0.312
5th year after diagnosis	10 (0.054%)	11 (0.03%)	1.82 [0.69 to 4.72]	0.171	0.282
10th year after diagnosis	14 (0.075%)	14 (0.038%)	2.00 [0.88 to 4.53]	0.071	0.139
15th year after diagnosis	24 (0.129%)	26 (0.07%)	1.85 [1.02 to 3.35]	0.035	0.071
20th year after diagnosis	27 (0.145%)	32 (0.086%)	1.69 [0.97 to 2.91]	0.052	0.102

#### 3.3.2. Agents Acting on the Renin–Angiotensin System (C09)

The ADHD group also demonstrated a higher use of medications in this category across all time points of follow-up. Notably, statistical significance was observed at 5 years (*p* = 0.013, q = 0.029) with an OR of 2.20 [95% CI: 1.15–4.25]. At 15 years, this trend became stronger (*p* = 0.007, q = 0.016), and at 20 years, the odds ratio slightly decreased to 1.39, with the significance marginally missed (*p* = 0.059, q = 0.113). Details are presented in Table 4.

**Table 4:** Agents Acting on the Renin–Angiotensin System Prescribed Following Initial Diagnosis Over a 20-Year Period (presented as number [%]).

C09 Agents Acting on The Renin–Angiotensin System	Case N = 18,558	Controls N = 37,116	OR (95%CI)	*p* Value	FDR BH
1st year after diagnosis	11 (0.059%)	8 (0.022%)	2.75 [1.01 to 7.88]	0.029	0.069
5th year after diagnosis	22 (0.119%)	20 (0.054%)	2.20 [1.15 to 4.25]	0.013	0.029
10th year after diagnosis	27 (0.145%)	40 (0.108%)	1.35 [0.80 to 2.26]	0.244	0.395
15th year after diagnosis	49 (0.264%)	57 (0.154%)	1.72 [1.15 to 2.57]	0.007	0.016
20th year after diagnosis	57 (0.307%)	82 (0.221%)	1.39 [0.97 to 1.98]	0.059	0.113

#### 3.3.3. Diuretics (C03)

The utilization of diuretics increased notably in the ADHD group over time. By the 15-year follow-up, there was a statistically significantly higher use compared to controls (*p* = 0.0028, q = 0.0067), with an OR of 1.77 [95% CI: 1.21–2.60]. This trend persisted into the 20-year follow-up (*p* = 0.003, FDR = 0.007), with a consistent OR of 1.73 [95% CI: 1.20–2.50]. Details are presented in Table 5.

**Table 5:** Diuretics Prescribed Following Initial Diagnosis of ADHD Over a 20-Year Period (presented as number [%]).

C03 Diuretics	Case N = 18,558	Controls N = 37,116	OR (95%CI)	*p* Value	FDR BH
1st year after diagnosis	4 (0.022%)	5 (0.013%)	1.60 [0.32 to 7.44]	0.493	0.691
5th year after diagnosis	7 (0.038%)	12 (0.032%)	1.17 [0.39 to 3.21]	0.809	0.949
10th year after diagnosis	11 (0.059%)	18 (0.048%)	1.22 [0.52 to 2.73]	0.694	0.816
15th year after diagnosis	54 (0.291%)	61 (0.164%)	1.77 [1.21 to 2.60]	0.003	0.007
20th year after diagnosis	58 (0.313%)	67 (0.181%)	1.73 [1.20 to 2.50]	0.003	0.007

#### 3.3.4. Stimulants

Table 6 and Table 7 present data on the prescription of stimulant medications among ADHD cases and controls. As expected, all individuals with an ADHD diagnosis had at least one fulfilled prescription for either methylphenidate or amphetamine, consistent with current treatment guidelines. Interestingly, a small proportion of the control group also received prescriptions for these medications. This may reflect off-label use for other conditions, such as narcolepsy or depression, or a temporary prescription issued for diagnostic purposes. In Israel, it is a common clinical practice to prescribe a single dose of a stimulant to assess its effect during computerized performance tests such as TOVA or MOXO, which are often conducted in two stages: one without medication and one following administration of a stimulant. Numerical details are presented in Table 6.

## 4. Discussion

The present study aimed to comprehensively investigate the association between ADHD and EH within a pediatric population. Our findings indicate a significant correlation between ADHD and elevated rates of EH, aligning with prior research that has suggested that ADHD is linked to various metabolic and cardiovascular disorders [15]. In addition to the increased prevalence of recorded diagnoses of EH among individuals with ADHD, we also observed higher rates of prescriptions for medications commonly used to treat EH. This dual finding strengthens the validity of our outcome measures and suggests that these were not incidental diagnoses but instead associated with meaningful hypertension that required active pharmacological management.

The novel finding of the present study of higher prevalences of the diagnosis of EH during the 20-year follow-up of children with ADHD, compared to well-matched controls, confirms the increased prevalence of hypertension in children with ADHD. This association can be attributed to several factors, including the potential adverse effects of ADHD medications, particularly stimulants, which can elevate heart rate and blood pressure [3,13]. Our dataset did not allow for precise sequencing of ADHD diagnosis, stimulant initiation, and hypertension coding, which limits our ability to fully disentangle the effects of ADHD itself from those of stimulant treatment. Unfortunately, quantifying stimulant exposure in real-world settings is highly challenging. Many individuals use these medications intermittently, often on an as-needed basis—such as before examinations or specific tasks—and may purchase a single prescription that lasts for months or even a year. This irregular usage pattern makes it difficult to accurately capture cumulative exposure or establish dose–response relationships using administrative data.

Furthermore, behavioral factors associated with ADHD, such as impulsivity and difficulties with emotional regulation, may influence lifestyle choices and health behaviors, indirectly increasing the risk of hypertension. The association between ADHD and hypertension may also be related to shared metabolic pathways, as it has been shown that ADHD is associated with various somatic comorbidities, systemic inflammation, and immune dysregulation [16,17,18,19,20,21,22,23,24,25,26,27,28]. Further research is needed to elucidate the complex interplay of these factors with the wish to identify potential targets for intervention.

Regarding the application to clinical practice, although the prevalence of hypertension in children with ADHD is very low, the hypertension appears already in childhood and persists for many years and is therefore of clinical significance. We think that the study results highlight the need to consider monitoring blood pressure in children diagnosed with ADHD, particularly those undergoing stimulant medication treatment. Healthcare providers should consider regular cardiovascular evaluations for pediatric patients with ADHD to mitigate potential risks and ensure early intervention if hypertension develops.

**Strengths and limitations**

This study has several strengths that enhance its contribution to the understanding of the association between ADHD and EH in the pediatric population. The nationwide study included a substantial number of participants and employed a cohort study design with prolonged follow-up, which provides robust statistical power, reduces the risk of selection bias, enhances the generalizability of the findings, and improves the representativeness of the study. The careful matching of cases and controls on key variables, such as age, gender, socioeconomic status, ethnicity and BMI, helps minimize the influence of confounding factors.

This study also has certain limitations that should be considered when interpreting the findings. The prevalence of EH observed in our study was lower than the estimates reported in the literature. This discrepancy likely reflects our reliance on formal diagnostic coding (ICD-9/10) rather than direct blood pressure measurements. Physicians may have delayed assigning a diagnosis of EH in children and adolescents, resulting in under documentation in electronic medical records, despite elevated blood pressure readings, and starting anti-hypertensive medications. Also, the diagnosis of EH in our study was based solely on ICD-9/10 diagnostic codes, as the clinical blood pressure measurements were not available for all patients, with the appropriate percentiles, which may limit the diagnostic precision. In the HMO setting, blood pressure is typically measured during routine clinical visits; however, not all patients may have undergone regular assessments, and some cases of hypertension may remain undiagnosed or undocumented. However, the matched control group overcomes the measurement bias and underdiagnosis. Another serious limitation was that, although most individuals with ADHD in our cohort received prescriptions for psychostimulant medications, primarily methylphenidate or mixed amphetamine salts, we were unable to determine whether the subgroup that developed hypertension had a higher exposure to these medications compared to those without hypertension. A key limitation is that our dataset did not capture medication dosage, titration patterns, or continuity of specific antihypertensive agents over time. We were able to report only the proportion of treated children at each interval, without insight into individual treatment regimens. However, the availability of comprehensive prescription records over a 20-year period allowed us to capture long-term trends in medication utilization across major antihypertensive classes, which strengthens the validity of our findings and provides valuable real-world insights into treatment patterns in pediatric ADHD populations.

Although the study controlled for major potential confounders, there may be other unmeasured/unknown factors that could contribute to the observed association between ADHD and EH, such as lifestyle, family history, or cardiometabolic risk clustering. A surveillance bias is possible, as physicians might have followed more closely the children with ADHD than the controls, which may increase the likelihood of hypertension being diagnosed and recorded compared to controls. Another limitation relates to our analytic approach. We used logistic regression at fixed intervals rather than survival analysis because precise event dates were unavailable; thus, time-to-event modeling was not feasible. While this method allowed us to estimate odds ratios at clinically meaningful horizons, it does not account for varying follow-up times and censoring, which may introduce bias. The study population was drawn from a single HMO in Israel, which may limit the generalizability of the findings to other populations with different healthcare systems or demographic characteristics. Future research should examine interactions between age, comorbidities, and medication exposure to better understand the multifactorial nature of hypertension risk in children with ADHD. This study may serve as a foundation for further investigations in diverse populations, ideally using prospective designs.

## 5. Conclusions

Our findings demonstrate a statistically significant association between ADHD and the subsequent clinically diagnosed EH and antihypertensive therapy. Regarding the retrospective nature of our study, with its inherent limitations, our findings should be interpreted as exploratory signals rather than definitive evidence. Future longitudinal studies are warranted to validate the current findings and explore the pathogenetic pathways underlying this association, including the effect of psychostimulant medications, and the potential roles of chronic inflammation, behavioral dysregulation. Clinical awareness of this comorbidity is suggested to enhance early screening and preventive strategies among youth with ADHD, addressing both neurodevelopmental and cardiovascular health in pediatric patients.

## Figures and Tables

**Table 1:** Demographic and clinical variables of the ADHD-diagnosed children and the matched control group.

		AHDH Group	Control Group	*p* Value	OR [95% CI]
Number		18,558	37,116		
Gender, n (%)	Male	11,777 (63.5%)	23,553 (63.5%)	0.999	1.00 [0.96 to 1.04]
	Female	6781 (36.5%)	13,563 (36.5%)	0.999	1.00 [0.96 to 1.04]
Age at diagnosis, year, mean (±SD)		8.38 (±2.71)	8.38 (±2.71)	0.979	
Age category, years, n (%)	≤9	13,581 (73.2%)	27,138 (73.1%)	0.871	1.00 [0.96 to 1.04]
	10–18	4977 (26.8%)	9978 (26.9%)	0.879	1.00 [0.96 to 1.04]
Sector, n (%)	General	10,280(55.3%)	20,488 (55.2%)	0.999	1.01 [0.97 to 1.04]
	Jewish Ultra-orthodox	6629(35.7%)	13,319 (35.9%)	0.708	0.99 [0.96 to 1.03]
	Arab	1649 (8.9%)	3309 (8.9%)	0.999	1.00 [0.94 to 1.06]
Geographic region, n (%)	Jerusalem	6891 (37.1%)	13,799 (37.2%)	0.919	1.00 [0.96 to 1.04]
	Center	4722 (25.4%)	9428 (25.4%)	0.918	1.00 [0.96 to 1.04]
	South	4356 (23.5%)	8705 (23.5%)	0.966	1.00 [0.96 to 1.04]
	North	2589 (14.0%)	5184 (14.0%)	0.969	1.00 [0.95 to 1.05]
Socioeconomic status, mean (SD)		7.92 (±3.94)	7.92 (±3.98)	0.928	1.00 [0.95 to 1.05]
Socioeconomic Status	Very low (1–3)	2717 (14.6%)	5432 (14.6%)	0.98	1.00 [0.96 to 1.05]
Category	Low (4–6)	3988 (21.5%)	7975 (21.5%)	0.943	1.00 [0.95 to 1.04]
	Medium (7–9)	3227 (17.4%)	6450 (17.4%)	0.911	1.00 [0.96 to 1.04]
	Medium-High (10–20)	6498 (35.0%)	13,020 (35.1%)	0.974	1.00 [0.95 to 1.06]
	Missing Data	2128 (11.5%)	4256 (11.5%)	0.97	1.00 [0.95 to 1.05]
BMI, mean (SD)		17.77 (±3.95)	17.47 (±3.94)	0.294	
Bronchial Asthma, n (%)		4666 (25.1%)	8393 (22.6%)	0.001	1.15 [1.10 to 1.20]
Diabetes Mellitus, n (%)		61 (0.33%)	130 (0.35%)	0.701	0.94 [0.68 to 1.28]
Epilepsy, n (%)		306 (1.65%)	294 (0.79%)	0.001	2.10 [1.78 to 2.48]
Physical activity	Missing	16,428 (88.52%)	32,694 (88.09%)	0.1355	1.04 [0.99 to 1.10]
Category	None	217 (1.17%)	488 (1.31%)	0.1593	0.89 [0.75 to 1.05]
	Occasionally	767 (4.13%)	1597 (4.30%)	0.3607	0.96 [0.88 to 1.05]
	1–3 h weekly	847 (4.56%)	1678 (4.52%)	0.829	1.01 [0.93 to 1.10]

**Table 2:** The prevalence of the diagnosis of essential hypertension among ADHD cases vs. non-ADHD controls (presented as number [%]).

Diagnosis of— Hypertension, Essential, (ICD-9 Code 401)	Cases N = 18,558	Controls N = 37,116	OR (95%CI)	*p* Value	FDR BH
1 year after ADHD diagnosis	2 (0.011%)	3 (0.008%)	1.33 [0.11 to 1.64]	1	1
5 years after ADHD diagnosis	19 (0.102%)	12 (0.032%)	3.17 [1.46 to 7.16]	0.002	0.035
10 years after ADHD diagnosis	22 (0.119%)	15 (0.04%)	2.94 [1.45 to 6.09]	0.001	0.025
15 years after ADHD diagnosis	39 (0.21%)	42 (0.113%)	1.86 [1.17 to 2.95]	0.006	0.097
20 years after ADHD diagnosis	47 (0.253%)	49 (0.132%)	1.92 [1.26 to 2.93]	0.002	0.028

**Table 6:** Methylphenidate Prescribed Following Initial Diagnosis of ADHD Over a 20-Year Period (presented as number [%]).

N06BA04 Methylphenidate	Case N = 18,558	Controls N = 37,116	OR (95%CI)	*p* Value	FDR BH
1st year after diagnosis	12,214 (65.815%)	190 (0.512%)	377.08 [318.73 to 434.68]	<0.0001	<0.0001
5th year after diagnosis	14,051 (75.714%)	721 (1.943%)	157.72 [144.90 to 171.60]	<0.0001	<0.0001
10th year after diagnosis	14,278 (76.937%)	1012 (2.727%)	118.93 [110.62 to 127.74]	<0.0001	<0.0001
15th year after diagnosis	13,894 (74.868%)	975 (2.627%)	110.32 [102.50 to 119.13]	<0.0001	<0.0001
20th year after diagnosis	14,542 (78.36%)	1202 (3.238%)	108.31 [101.11 to 115.34]	<0.0001	<0.0001

**Table 7:** Amphetamine Prescribed Following Initial Diagnosis of ADHD Over a 20-Year Period (presented as number [%]).

N06BA01 Amphetamine	Case N = 18,558	Controls N = 37,116	OR (95%CI)	*p* Value	FDR BH
1st year after diagnosis	1598 (8.611%)	17 (0.046%)	205.54 [128.34 to 353.29]	<0.0001	<0.0001
5th year after diagnosis	4260 (22.955%)	106 (0.286%)	103.69 [85.53 to 127.96]	<0.0001	<0.0001
10th year after diagnosis	5095 (27.454%)	168 (0.453%)	83.23 [71.20 to 97.94]	<0.0001	<0.0001
15th year after diagnosis	4515 (24.329%)	120 (0.323%)	99.34 [82.28 to 119.59]	<0.0001	<0.0001
20th year after diagnosis	5157 (27.789%)	178 (0.48%)	79.85 [68.55 to 93.78]	<0.0001	<0.0001

## Data Availability

Data supporting the findings of this study are available upon reasonable request, subject to the regulatory requirements of Leumit Health Services and Israeli Ministry of Health directives.
